# Pilot Evaluation of Possible Airborne Transmission in a Geriatric Care Facility Using Carbon Dioxide Tracer Gas: Case Study

**DOI:** 10.2196/37587

**Published:** 2022-12-30

**Authors:** Yo Ishigaki, Shinji Yokogawa, Yuki Minamoto, Akira Saito, Hiroko Kitamura, Yuto Kawauchi

**Affiliations:** 1 Graduate School of Informatics and Engineering University of Electro-communications Tokyo Japan; 2 Info-powered Energy System Research Center University of Electro-communications Tokyo Japan; 3 School of Engineering Tokyo Institute of Technology Tokyo Japan; 4 Department of Clinical Laboratory Medicine Miyagi Anti-Tuberculosis Association Miyagi Japan; 5 Occupational Health Training Center University of Occupational and Environmental Health Fukuoka Japan

**Keywords:** nursing home, care home, airborne transmission, ventilation frequency, air change rate, ACR, computational fluid dynamics, CFD, mobile phone

## Abstract

**Background:**

Although several COVID-19 outbreaks have occurred in older adult care facilities throughout Japan, no field studies focusing on airborne infections within these settings have been reported. Countermeasures against airborne infection not only consider the air change rate (ACR) in a room but also the airflow in and between rooms. However, a specific method has not yet been established by Japanese public health centers or infectious disease–related organizations.

**Objective:**

In April 2021, 59 COVID-19 cases were reported in an older adult care facility in Miyagi, Japan, and airborne transmission was suspected. The objective of this study was to simultaneously reproduce the ACR and aerosol advection in this facility using the carbon dioxide (CO_2_) tracer gas method to elucidate the specific location and cause of the outbreak. These findings will guide our recommendations to the facility to prevent recurrence.

**Methods:**

In August 2021, CO_2_ sensors were placed in 5 rooms where airborne infection was suspected, and the CO_2_ concentration was intentionally increased using dry ice, which was subsequently removed. The ACR was then estimated by applying the Seidel equation to the time-series changes in the CO_2_ concentration due to ventilation. By installing multiple sensors outside the room, advection outside the room was monitored simultaneously. Aerosol advection was verified using computer simulations. Although the windows were closed at the time of the outbreak, we conducted experiments under open-window conditions to quantify the effects of window opening.

**Results:**

The ACR values at the time of the outbreak were estimated to be 2.0 to 6.8 h^−1^ in the rooms of the facility. A low-cost intervention of opening windows improved the ventilation frequency by a factor of 2.2 to 5.7. Ventilation depended significantly on the window-opening conditions (*P* values ranging from .001 to .03 for all rooms). Aerosol advection was detected from the private room to the day room in agreement with the simulation results. Considering that the individual who initiated the infection was in the private room on the day of infection, and several residents, who later became secondarily infected, were gathered in the day room, it was postulated that the infectious aerosol was transmitted by this air current.

**Conclusions:**

The present results suggest that secondary infections can occur owing to aerosol advection driven by large-scale flow, even when the building design adheres to the ventilation guidelines established in Japan. Moreover, the CO_2_ tracer gas method facilitates the visualization of areas at a high risk of airborne infection and demonstrates the effectiveness of window opening, which contributes to improved facility operations and recurrence prevention.

## Introduction

### Background

COVID-19 should be prevented in health care and long-term care facilities because of the high risk of mortality [1]. In Japan, a series of COVID-19 outbreaks has been reported in nursing care facilities [2]. However, no field epidemiology case studies have assessed airborne infection by measuring the air change rate (ACR) and airflow in Japanese older adult care facilities.

Ventilation plays an important role in controlling the airborne transmission of COVID-19, with mass transmission of its etiologic pathogen, SARS-CoV-2, reported in poorly ventilated rooms. This was demonstrated in Ishigaki et al [3], which noted that ventilation was impeded by excessive shielding of an office space with plastic sheeting where a 7-person outbreak occurred. Li et al [4] reported a 9-person outbreak that occurred locally in a poorly ventilated restaurant, and Jang et al [5] reported that 112 people were infected in a small fitness studio with very poor ventilation in South Korea. Furthermore, Menzies et al [6] reported that in various hospital settings, an average of less than 2 ventilation cycles per hour (ACR per hour) in examination rooms represented a determinant of secondary tuberculosis transmission, that is, tuberculin conversion. On the basis of this study, the Centers for Disease Control and Prevention (CDC) in the United States established a standard for negative pressure room ventilation to isolate patients with infectious diseases, which included an ACR recommendation of 6 per hour (for existing buildings) to 12 per hour (for new buildings) with a safety factor [7]. Subsequently, based on the CDC standard of 12 (h^−1^) ACR, the World Health Organization (WHO) established a standard of 576 m^3^/hour per person, with a doubled safety factor, for natural ventilation in health facilities treating patients with infectious diseases, assuming that each patient occupies a space of 4 × 2 × 3 m^3^ [8]. In Japan, the Ministry of Health, Labor and Welfare (MHLW) has suggested a similar value of 576 m^3^/hour per person for health facilities. Furthermore, the MHLW has recommended a ventilation rate of 30 m^3^/hour per person in general commercial facilities to prevent indoor aerosol transmission of COVID-19 [9].

Fine or dry droplets can remain airborne for several minutes to several hours [10-13]. Therefore, secondary infections can occur when these infectious aerosols are transported by air currents, regardless of the distance from the infected person. Airborne transmission of COVID-19 was suspected in a shopping mall in China, as shoppers not in direct contact with each other became infected at the same time [14]. In Australia, a secondary infection occurred in a church with minimal ventilation, from a choir to 12 attendees, with a reported airborne distance of up to 15 m [15]. Moreover, 14 people were infected in a wide range of seats evenly distributed from rows 7 to 29 on both sides of a plane during a domestic short aisle flight in Japan [16]. Hence, when designing countermeasures against airborne infection, it is necessary to consider not only the number of times a room is ventilated but also the airflow within and between rooms.

Pathogens such as *Mycobacterium tuberculosis* (etiologic pathogen of tuberculosis), measles, and varicella-zoster virus (chicken pox) are airborne [17]. The long-range transmission of infectious aerosols containing these pathogens can be controlled by buoyancy because of temperature differences and airflow control by natural winds and fans [18]. However, several reports have indicated that the effective reach of airborne COVID-19 transmission is at least 2-10 m. For instance, Anchordoqui et al [19] used computer simulations to analyze the aerodynamic properties of particulate matter containing SARS-CoV-2 and noted that it could propagate farther than the recommended social distance of 1.8 m. Morawska et al [20] reported multiple cases of propagation beyond a distance of 1-2 m. Hunziker [21] focused on the behavior of aerosols in an air-conditioned hospital room and reported aerosols with micron-order particle size propagating up to a distance of 5-6 m as jet passengers. However, according to Guven et al [22], micron-order particles propagate over long distances of 2-8 m. Moreover, Anderson et al [23] found that airborne viruses can remain active for up to 27 hours depending on the conditions of temperature and humidity, whereas infectious aerosols can travel up to 7.0-8.2 m. More specifically, infectious aerosols, or gas clouds, formed by sneezing can reach a distance of 7-8 m and remain in the air for hours, depending on the ventilation system [24]. In addition, Lima et al [25] surveyed 10 studies and concluded that aerosol particles containing viruses can reach distances of up to 10 m and survive for several hours in air. Furthermore, Azimi et al [26] investigated a large outbreak on a cruise ship in which 712 of 3711 crew members and passengers were infected, concluding that reinhalation of infectious aerosols was the primary route of transmission.

### Goal of This Study

In this study, an outbreak site of SARS-CoV-2 was investigated in a nursing home in Miyagi Prefecture, Japan, to measure the ACR and visualize the behavior of infectious aerosol distribution using a carbon dioxide (CO_2_) tracer gas method—that can estimate the actual ACR of a room and is used to prevent airborne transmission of COVID-19 [27]—at various window-opening conditions. Aerosol advection originating from the room was quantified by simultaneously monitoring the tracer gas leaking from the room. In addition, the aerosol distribution was analyzed via computational fluid dynamics (CFD) based on a 3D model of the facility to ensure that the 3D behavior corresponded with the measured CO_2_ tracer gas results. CFD simulations are effective for detailing the airborne behavior of infectious aerosols [28]. However, its implementation requires specialized knowledge, detailed surveying, and computational resources. In contrast, the CO_2_ tracer gas method can be implemented with dry ice and a sensor, and if its effectiveness is confirmed, it can be used in numerous facilities to prevent recurrent outbreaks.

The primary purpose of this study was not to identify the direct cause of the outbreak but to identify the factors responsible for, and the origin locations of, outbreaks in older adult care facilities in terms of ACR and aerosol dispersion. Furthermore, this study seeks to establish a CO_2_ tracer gas method that can simultaneously monitor ventilation and aerosol behavior in hospitals and older adult care facilities to contribute to the prevention of recurrent airborne infections.

## Methods

### Facility Overview

The older adult care facility investigated in this study was located in Miyagi Prefecture, Japan, where 59 cases were reported in the same building from April to May 2021. Of these, 36 were users of the facility (29 residents and 7 daily visitors) and the other 23 were facility staff members. As 2 positive cases of infection were obtained on the first day using polymerase chain reaction (PCR) tests, and the subsequent PCR tests performed 2 days later further confirmed the positivity of 14 people on the same floor, airborne transmission was strongly suspected to be responsible for the propagation of the infection. Other causes include contact and droplet infections, making it almost impossible to identify a direct cause. However, as masks and face shields were worn and hand sanitizers were used by staff members who spread the secondary infection, and as the infection spread in a short period among those who were not in close contact with each other, it is logical to suspect airborne transmission in these circumstances. Multimedia Appendix 1 summarizes the time course of the emergence of infected patients in April. After the outbreak, the Disaster Medical Assistance Team was dispatched to the zone and critically ill patients were hospitalized, after which the infection was considered under control. The field survey reported in this study was conducted in August 2021.

**Figure 1:**
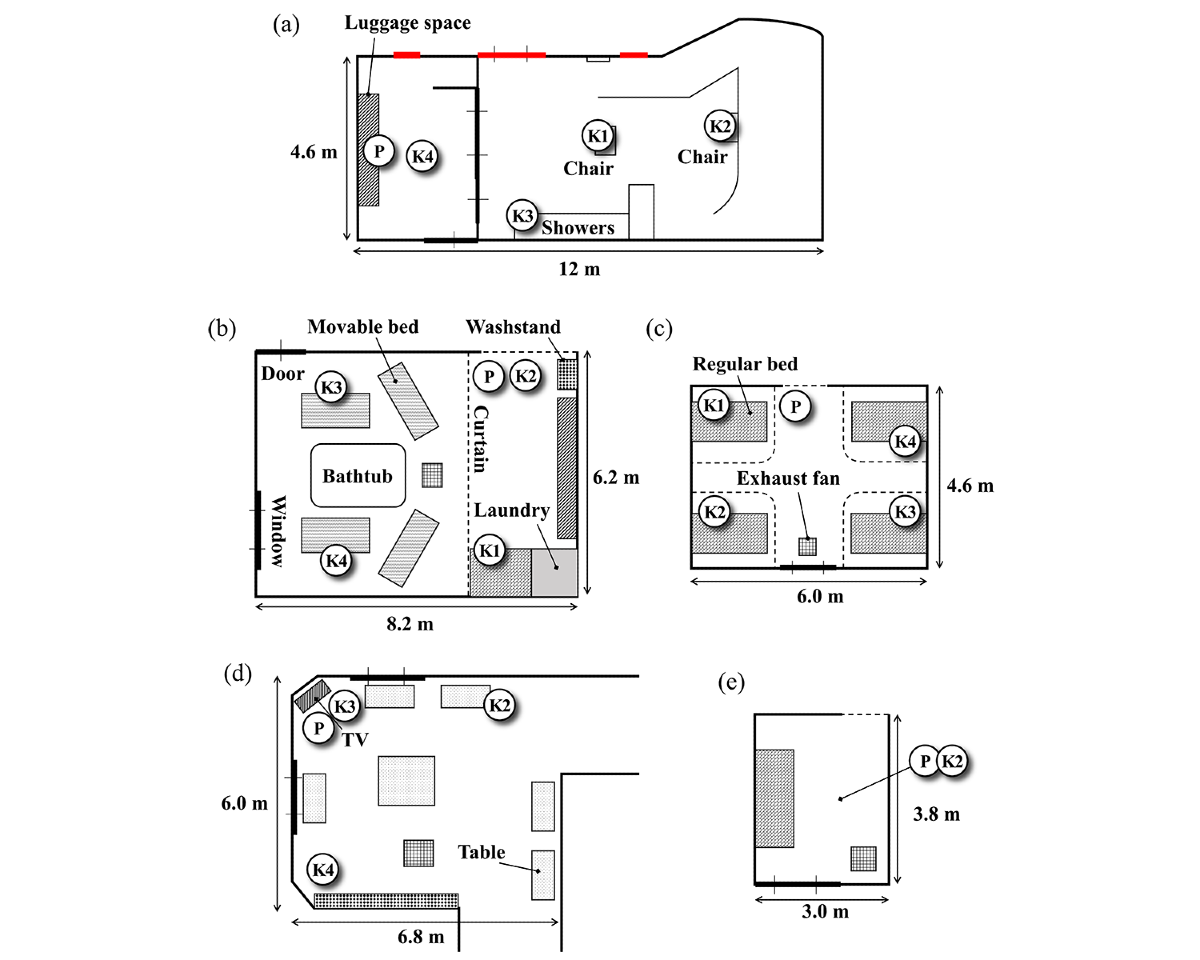
Floor plan of each room investigated during the primary measurement. The locations of sensors P and K1-K4 are also shown. (a) Regular bathroom, (b) nursing bathroom, (c) shared room, (d) day room, and (e) private room. The ceiling heights of these rooms were 2.6, 2.4, 2.7, 2.4, and 2.4 m, respectively. K: TR-76Ui sensor used; P: SCD-30 sensor used; TV: television.

On the basis of the interviews with facility staff, the following five separate areas in the building, where large-scale secondary infection occurred, were extensively studied: (1) regular bathrooms, (2) nursing bathrooms, (3) shared rooms, (4) private rooms, and (5) day rooms, as shown in Figure 1. Residents used both regular and nursing bathrooms, and staff members accompanied them for assistance. The risk of aerosol infection is considered to increase in bathrooms, as neither the residents nor staff wear a mask because of the high-humidity environment. Furthermore, a care recipient using a nursing bathroom requires high-level care, and the staff must talk to them while making contact, which is expected to increase the risk of transmission. The shared room had a maximum of 4 beds, 3 of which were for residents, whereas the other was unused at the time of the outbreak. Although residents were instructed to wear masks at all times, it was difficult to enforce them under certain conditions, such as dementia. A private room is the one for a single resident; therefore, the risk of aerosol infection is relatively low. It was segregated from the corridor using a curtain. The first resident who tested positive for COVID-19 was within a private room next to the day room; subsequently, a mass infection outbreak occurred. The day room is a place for relaxation; has free access for all residents and other visitors temporarily visiting the facility between 6:00 AM and 8:00 PM daily; and is furnished with chairs, tables, and televisions. As the day room is frequently used by multiple people, including care recipients and staff, the risk of infection is expected to be relatively high. These rooms were first investigated individually during the primary measurements, as described later. Therefore, the layout of these rooms in the entire floor plan was irrelevant. Furthermore, several transmission routes were involved in the studied mass infection outbreak event. This study focused on airborne transmission via aerosols; however, other transmission routes, such as direct contact, were not excluded.

### Ethics Approval

This study was approved by the Ethics Committee on Experiments on Human Subjects at the University of Electro-Communications, Chofugaoka 1-5-1, Chofu, Tokyo, Japan (approval number: 21,005).

### Measurements

On-site measurements were conducted under the guidance of an industrial physician to ensure safety while confirming that the results of the PCR test of all residents, staff, and researchers were negative, and safety measures, such as the use of personal protective equipment and disinfection, were taken. In this study, the following 2 types of experiments were performed: primary and secondary measurements.

#### Primary Measurement

In the primary experiment conducted on August 13, 2021, the tracer gas method was used to measure the ACR in 5 areas. CO_2_, which was obtained by vaporizing dry ice in the study room, was used as the tracer gas. Two types of nondispersive infrared–type CO_2_ sensors were used. The first was a mobile CO_2_ sensor (Yaguchi Electric Corporation) equipped with a nondispersive infrared sensor SCD-30 (Sensirion AG). Any measurements performed with this sensor are hereafter designated “P.” The second sensor was a TR-76Ui (T&D Corporation), designated “K,” with an index of measurement locations. A total of 5 sensors (1 P sensor and 4 K sensors, K1-K4) were used during the measurements. Figure 1 shows the arrangement of the sensors in each room. All the sensors were placed at a height of approximately 1 m from the ground.

Measurements were conducted in each room as follows:

1. The CO_2_ sensors were installed at the locations shown in Figure 1, and the measurements were initiated.
2. The mechanical ventilation system in the room of interest was turned off, and the windows and doors, if any, were closed to create a closed room.
3. Dry ice was placed in the room of interest to achieve a CO_2_ concentration sufficiently high compared with the background (approximately 400 ppm). The concentration must be at least 2000 ppm but should not exceed the permissible concentration of 5000 ppm (at 8 h of exposure), as specified by the Japanese Industrial Safety and Health Law. In addition, a blower was used to sufficiently mix the room air with the generated CO_2_ gas, as the gas evaporated from the dry ice yielded a low temperature and tended to remain close to the floor.
4. When the CO_2_ concentration was sufficiently increased, dry ice was removed from the room, and the mechanical ventilation system and conditions of the windows and doors were set according to the target measurement conditions. The time point was denoted as t_*0*_.
5. The room was immediately vacated to avoid CO_2_ addition from breathing. This time point was defined as the start of ventilation (t_*sta*_)
6. CO_2_ concentration was monitored remotely from outside the room.
7. Once the CO_2_ concentration decreased sufficiently, the measurement was completed; this time point was denoted as t_*end*_. The time-series measurement data from t_*sta*_ to t_*end*_ were saved for analysis and used to estimate ACR.
8. If the CO_2_ concentration remained sufficiently high, step 3 could be omitted, and we proceeded to step 4.

The measured CO_2_ concentration data were processed to calculate the ACR in each room. The Seidel equation is as follows:



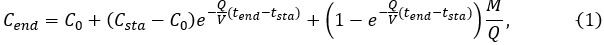



where *C_end_* is the CO_2_ concentration (ppm) at *t = t_end_, C*_0_ is the steady-state value of the CO_2_ concentration in the absence of pollution (ppm), *C_sta_*, is the concentration (ppm) at *t = t_sta_*, *V* is the room volume (m^3^), *Q* is the ventilation rate (m^3^/h), and *M* is the rate of pollutant generation (ppm m^3^/h). The measurement start and end times, *t_sta_* and *t_end_*, are in hours. Furthermore, *C*_0_ was assumed to be 400 ppm. Because the room was vacant during the measurement, *M* = 0 in equation 1, which means the following:



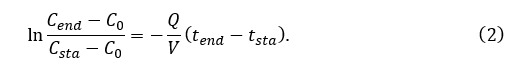



The ACR value, calculated as *Q / V* (1/h), was the decrease in the CO_2_ concentration from *t_sta_* until *t_end_*.

#### Secondary Measurement

**Figure 2:**
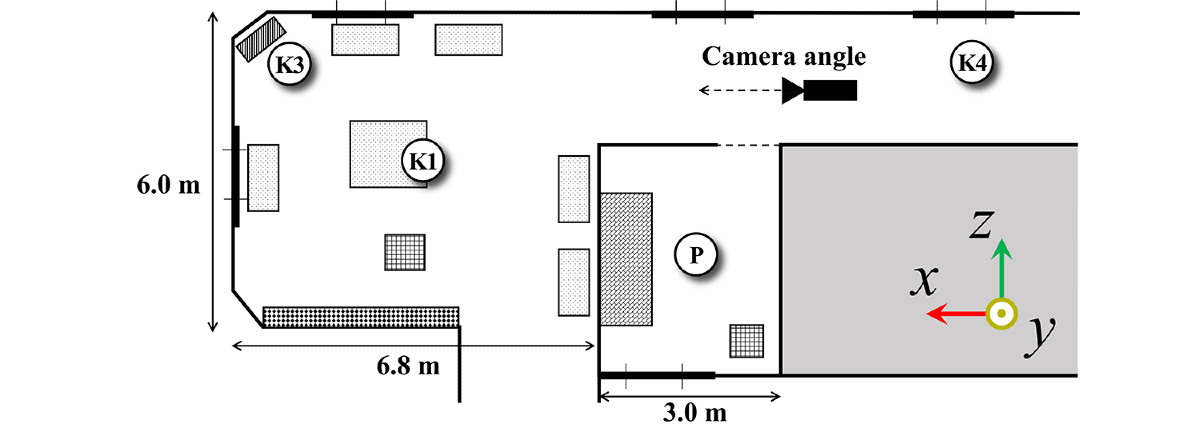
Floor plan including the private and day rooms connected by the short corridor and sensor locations for the secondary measurement. The orientation axes for the numerical simulation are shown. The first COVID-19–positive resident was found in a private room next to the day room. The locations of different sensors P, K1, K3, and K4 are also shown. K: TR-76Ui sensor used; P: SCD-30 sensor used.

In addition to measuring the 5 rooms individually, secondary measurements (Figure 2) were performed on August 13, 2021, to investigate the effect of the interplay between airflow in the private and day rooms, as these rooms are spatially connected via a 2-3 m long corridor. This dynamic fluid aspect suggests that aerosols leaked from the private room to the day room. A resident who was COVID-19–positive in the early stage of the mass outbreak was isolated in a private room. For secondary measurement, the sensors were placed as shown in Figure 2. The private room was filled with CO_2_ gas, measurement steps 1 to 6 were followed, and gas leakage from the private room to the day room was assessed.

**Figure 3:**
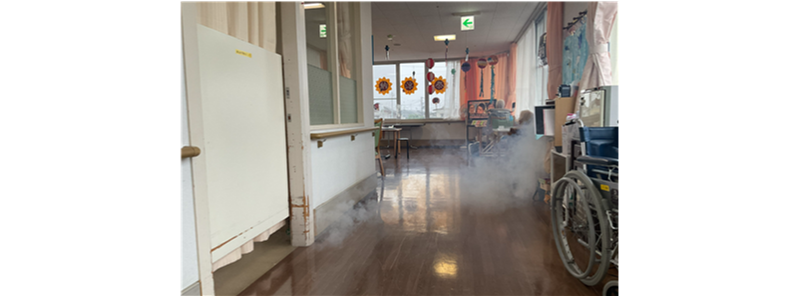
Direct photograph of the carbon dioxide smoke leaking from the private room of the first COVID-19–positive patient into the corridor leading to the day room containing multiple lounging tenants (taken from the camera angle shown in Figure 2).

Figure 3 provides a photograph of the CO_2_ smoke leaking from the private room shown in Figure 2 to the corridor leading to the day room, captured from the camera angle shown in Figure 2. A substantial amount of CO_2_ smoke leaked from the gap between the curtain and the floor and advected toward the ceiling. By the time it reached the day room, a high concentration of CO_2_ smoke was observed near the head height of a person sitting in a wheelchair.

### Numerical Simulation

Given that the spatiotemporal distribution of CO_2_ concentration is not readily determined from the secondary measurement experiment, a numerical simulation of gas leakage from the private rooms to the day rooms was performed. This simulation corresponded to a secondary measurement. The simulation was performed using FlowSquare+ (version 2021R1.0, Nora Scientific Co. Ltd), which solves the transport equations for mass (density, *ρ*):



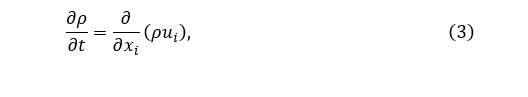



for momentum *ρu_i_*:



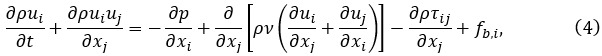



for energy (temperature, *T*):



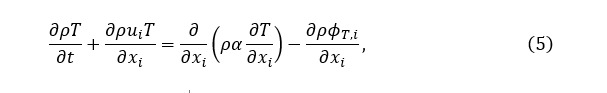



and for the mass fraction of the CO_2_ gas generated by the dry ice *Y_CO2_*:



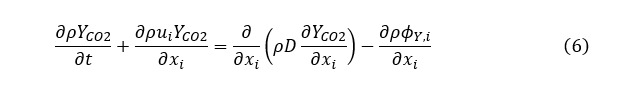



in a large eddy simulation context. The subscript indexes *i=*, 2, and 3 correspond to the directions *x, y,* and *z*, respectively; *v, α,* and *D* are the kinematic viscosity, thermal diffusivity, and molecular diffusivity, respectively; and *ρv* = 2.0 × 10^-6^ (kg/m/s), *α = v / Pr*, and *D = v / Sc*, where the Prandtl number (*Pr*) = 0.7 and the unity Schmidt number are considered. The subgrid-scale stress tensor, *τ_ij_*, and scalar fluxes, *ϕ_T,I_,* and *ϕ_Y,I_,* were modeled based on static Smagorinsky and gradient diffusion models, respectively. The transport equations were discretized onto *Nx* × *Ny* × *Nz = 175* × *90* × *30* uniform mesh points using a second-order finite-difference scheme in space and advanced in time using the explicit Euler method. Only the advection term was calculated using the first-order upwind scheme. The domain dimensions were *Lx* × *Ly* × *Lz = 17.5* × *3.0* × *9.0* (m^3^). Physical boundaries that did not coincide with the Cartesian mesh were expressed using a second-order immersed boundary method.

The fluid in the entire domain was initialized at 18 °C and *Y_CO2_* = 0.0. Warm air supplied by the air conditioner in the room produced a temperature of 30 °C. The fluid velocity of the air conditioner installed in the private room was (*ux*, *uy*, *uz*) = (0, −1, 2) m/s, whereas that of the air conditioner installed in the day room was (0, −1, 1) m/s. The ventilation fan in the private room was turned off, whereas that in the air from the day room was discharged at velocities of (0, 10, and 0) m/s. These settings were based on measurement conditions, considering the situation during mass infection outbreaks. To mimic the aerosol dispersion from a COVID-19–positive person in a private room, a small inflow boundary was considered on the bed with a constant (CO_2_) gas flow at 36 °C at a velocity of (0, 0.707, 0.707) m/s; therefore, its magnitude was 1.0 m/s. Furthermore, the fluid flow issued at this inflow boundary yielded *Y_CO2_* = 1.0.

During the numerical simulation, measurement probes were located for P, K1, and K3, as in the measurements (Figure 2), which facilitated the validation of the simulation results by comparing the measured data. The measurement at K4 was not performed in the simulation because of its proximity to the computational boundary.

## Results

### ACR Estimation

Table 1 summarizes the ACR values calculated using equation 2 from the measured CO_2_ concentration for each sensor in each room, as shown in Figure 1. We conducted a factorial effect analysis using a general linear model with the estimated ACR as the objective variable and window-opening conditions, sensor location, and sensor model (P or K) as explanatory variables.



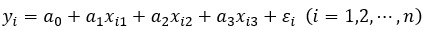



where *y_i_* is the ACR estimated from the experiments, *x_i1_* is the variable indicating the window conditions, *x_i2_* is the variable indicating the sensor locations (Figure 1), *x_i3_* is the variable related to the sensor model, a_0_, … ,a_3_ are the regression coefficients, and *ε_i_* is the error that is independent and identically distributed in normal distribution. As the explanatory variables are nominal measures, they were transformed into dummy variables for the analysis. This analysis, using a general linear model, can be regarded as factorial effect analysis with a 3-factor analysis of variance. JMP Pro 16.2.0 (SAS Institute Inc) was used for the analysis. The estimated ACR used in the analysis was 15 h^−1^ for the regular bathroom, 11 h^−1^ for the nursing bathroom, and 10 h^−1^ for the shared room.

**Table 1:** Air change rate (ACR) values and per capita ventilation volumes calculated based on the primary measurement results in each room shown in Figure 1.

Room and window-opening condition^a^			Measured ACR								Ventilation volume^b^ m^3^/person
		P^c^ (h^−1^)		K1^d^ (h^−1^)	K2 (h^−1^)	K3 (h^−1^)	K4 (h^−1^)	Ave.^e^ (h^–1^)	Ratio (r_ACR_)^f^		
**Regular bathroom**											
	Closed^g^	5.13		3.56	2.56	6.14	1.25	3.73	1.00^h^	56.7^i^	
	Open1^j^	1.00		1.00	7.99	3.81	1.00	5.90	1.58	70.6^i,k^	
	Open2^j,l^	1.00		5.38	7.5	1.00	11.9	8.25	2.21	84.0^i,k^	
	Open3^j,l,m^	9.41		9.37	9.62	9.77	11.2	9.86	2.65	125^i,k^	
**Nursing bathroom**											
	Closed^g^	2.38		1.56	3.17	2.55	2.27	2.39	1.00	36.8^i^	
	Open	16.6		11.8	11.0	16.1	18.9	14.9	5.74	211^i,k^	
**Shared room**											
	Closed^g^	3.67		1.05	2.34	1.09	1.81	1.99	1.00	38.0^i^	
	Open	4.74		10.4	7.40	6.68	7.20	7.29	3.59	136^i,k^	
**Private room**											
	Closed^g^	3.74		1.00	6.17	1.00	1.00	4.96	1.00	67.8^i^	
	Open	16.2		8.66	6.81	11.5	16.8	12.0	2.18	148^i,k^	
**Day room**											
	Closed^g^	7.79		1.00	4.26	6.73	8.47	6.81	1.00	30.6^i,k^	

^a^State of window opening and closing set in step 4 in the *Methods* section.

^b^Ventilation volume per person per hour calculated by multiplying the average air change rate (ACR) by the volume of the room and dividing it by room capacity.

^c^P: SCD-30 sensor.

^d^K: TR-76Ui sensor.

^e^Arithmetic mean of the calculated ACR values recorded by different sensors in each room.

^f^Ratio of ACR values between “Open” and “Closed” conditions.

^g^Conditions during mass infection outbreaks.

^h^Since “Closed” is the denominator in determining the ratio, it is expressed as 1.00 as a ratio to itself.

^i^Measured hourly ventilation volume compliant with the Ministry of Health, Labor and Welfare guidelines (2020).

^j^Two windows near the chairs (highlighted in red in Figure 1) were also opened.

^k^Measured hourly ventilation volume compliant with the Centers for Disease Control and Prevention guidelines (2003).

^l^A window near the luggage space (highlighted in red in Figure 1) was also opened.

^m^The windows facing the hallway outside the bathroom were additionally opened.

### Effects of Window-Opening

**Figure 4:**
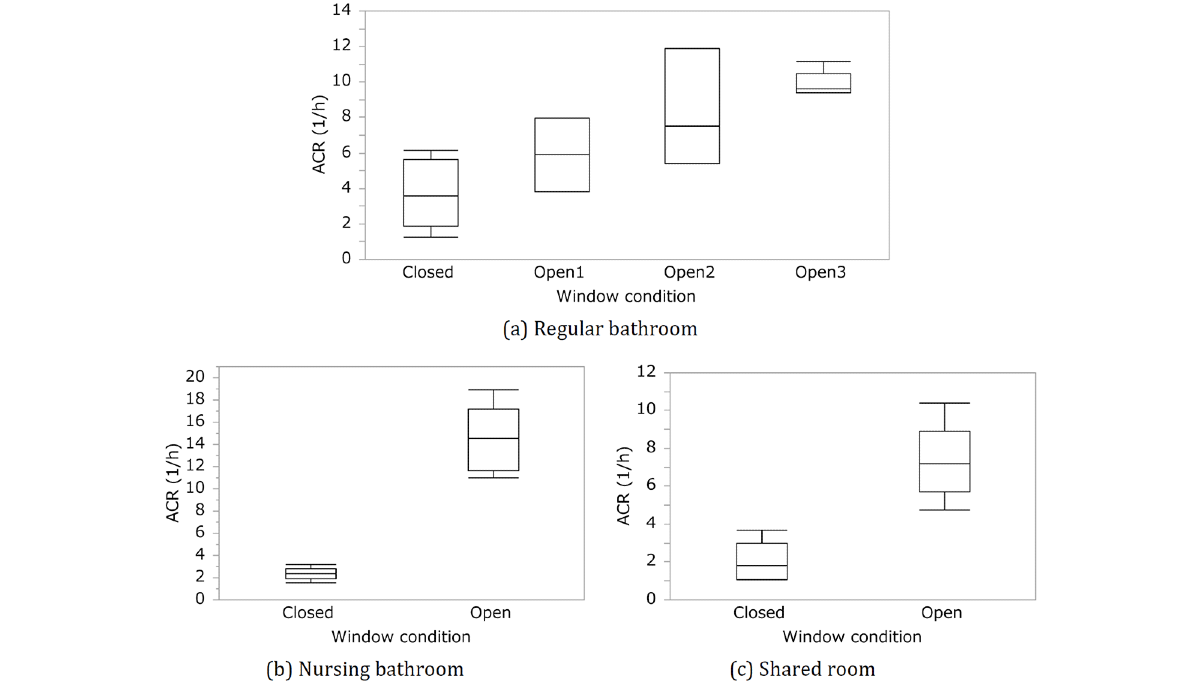
Box plots of estimated air change rates (ACRs) showing the effects of opening the window in a (a) regular bathroom, (b) nursing bathroom, and (c) shared room. The window condition agrees with the definition presented in Table 1.

The results showed that ventilation had a significant effect on window-opening conditions in all rooms (*P*=.027, .001, and .03 for the regular bathroom, nursing bathroom, and shared room, respectively). This is consistent with the box plots of the ACR merged with the sensor locations and models for the opening and closing of the windows, as shown in Figure 4. In contrast, no significant effect was observed at any of the sensor locations. Therefore, ventilation in the room was likely uniformly improved by opening the window in the regular and nursing bathrooms and the shared room. There was no significant dependence on the sensor model. It is well known that the analysis of variance is somewhat robust regarding normality (the population distribution of the observed values in each group is normally distributed) and equality of variance (the population variance of each group is equal). Consistent results were also obtained using Welch 2-tailed *t* test, which was used to test the hypothesis that 2 populations have equal means regarding 2 samples that may have unequal variances. Therefore, the analysis of the effect of window conditions is considered valid.

A more detailed analysis was conducted for the private room, where the first resident who tested positive for COVID-19 was staying. In the private room, the P and K2 sensors were installed close together because of the limited size of the room. Thus, we used a generalized linear mixed model (GLMM) to estimate and test the effects of ACR and window-opening conditions (with ventilation time and window-opening set as fixed effects) and the difference between sensor models (with the sensor model as a random effect). A GLMM was assumed for the model in equation 2 with the sensor model difference as a variable effect, and an analysis of the change in CO_2_ concentration with ventilation time was conducted. On the assumption of normal distributed random effects, 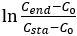, *y* can be expressed in the form of a GLMM as follows:

*y* = xβ + *Zu + e*
**(7)**

where *β* is the fixed effect, *u* and *e* are the normally distributed random effects, and *X* and *Z* are the design matrices. The fixed effect of the GLMM, which indicates a time-series improvement, can be summarized as *E(y) = Xβ. Zu* is the random effect of the individual variations. Here, *e* represents the intraindividual random effect. JMP (version 16.2) was used for the analysis using the maximum likelihood method. During the covariance parameter estimate of the random effect, the Wald *P* value of the sensor’s measurement was not significant (*P*=.96). Therefore, no difference in the sensor measurements was observed. However, the estimation of the fixed effects showed that the effects of ventilation time and window status were both significant, and their interaction was also highly significant (*P*<.001). The CO_2_ reduction (Q/V in equation 2) was 3.90/h without window opening and 11.52/h with window opening. In other words, it was confirmed that window opening improved the ACR by a factor of 3.

Although the windows were closed during the mass infection outbreak, even under these conditions, all rooms met the MHLW standard for ventilation (>30 m^3^/h per person) [6], suggesting that additional ventilation measurements were unnecessary. Furthermore, the *r_ACR_* values in Table 1 indicate that in all rooms, except the day room where the window-open experiment was not conducted, the ACR substantially improved by 2.2 to 5.7 times by opening the window to meet the CDC standard [4]. There was no significant dependence on the location or model of the sensor in all rooms where the window-open experiments were conducted, suggesting that the proposed method for estimating the ACR is efficient. Even under open-window conditions, no room met the WHO criteria [5]. However, because this facility is not classified as an infectious disease ward, this level of ventilation capacity is not strictly required.

### Fluid Advection

**Figure 5:**
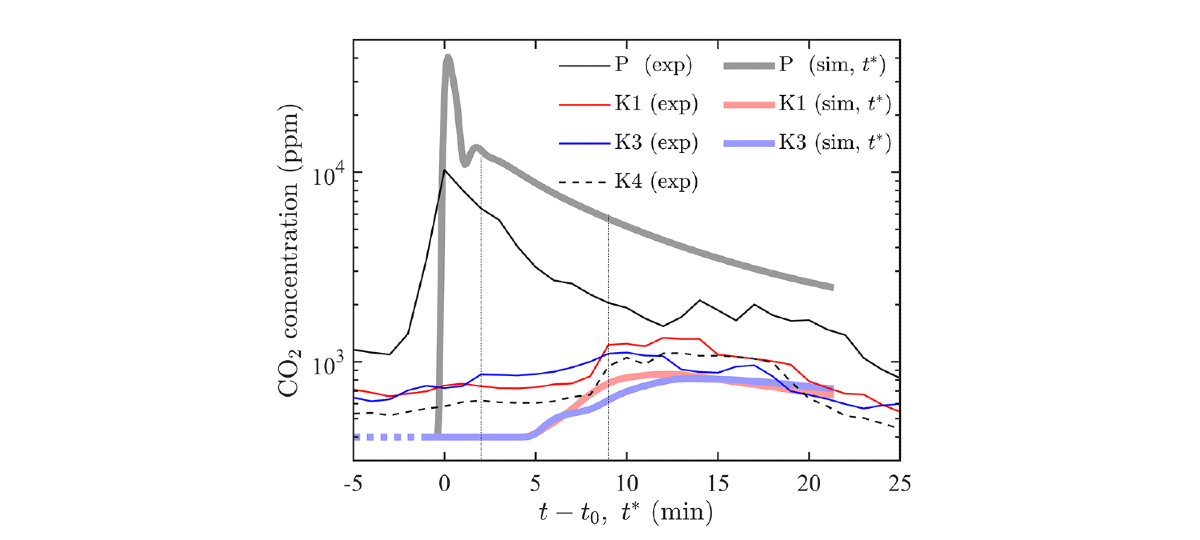
Temporal variation of the measured carbon dioxide (CO_2_) concentration during the secondary measurement (thin line) and the numerical simulation (thick line) at the sensor positions shown in Figure 2. Note that the modified time axis t* is used for comparison with the simulation results. The vertical lines indicate t – t_0_ (=t*) = 2 and t – t_0_ (=t*) = 9. K: TR-76Ui sensor used; P: SCD-30 sensor used.

The temporal variation of the measured CO_2_ concentrations in a larger area, including the private room and the day room (Figure 2), is shown in Figure 5. After the generation of CO_2_ gas was stopped at step 4 (as per the primary measurement subsection) at *t = t_0_*, the concentration at sensor P steadily decreased with time. Although the concentration of CO_2_ measured at K1 to K3 fluctuated, it exhibited a gradual increase toward *t – t_0_* = 9 minutes. The concentration at K3 was substantially higher than that of the other 2 K sensors in the early measurement stage (*t– t_0_* = 2 min), suggesting that the generated CO_2_ was predominantly advected from the private room by a large flow pattern rather than a simple diffusion process. This is also supported by the results shown in Figure 4, suggesting that ventilation performance solely depended on the window-opening condition, which dictates airflow. Later in the measurement (*t – t_0_* > 9 min), the CO_2_ concentrations at K1 and K4 surpassed that of K3, with a relatively steep slope in variation. This rapid change in the rate of concentration increase was due to advection, rather than diffusion.

The evolution of temporal CO_2_ concentrations for the positions of the numerical simulations at the positions of the P, K1, and K3 probes is shown in Figure 5. Despite attempts to mimic the environment in a nursing home facility, certain uncertainties in the experimental setup, such as the initial field and precise boundary conditions for ventilation or air conditioning devices, were unavoidable. Although this causes a discrepancy between the simulation results and measured data, the CO_2_ concentration obtained from the numerical simulation showed levels quantitatively similar to the measured values. Furthermore, the evolution of the CO_2_ concentration shows similar trends after multiplying the time axis of the simulation results by an a posteriori factor of 2.5, that is, t* = 2.5(*t_sim_* – *t_sim,0_*), where the subscript “sim” denotes the physical time in the simulation.

Regarding the evolution of the CO_2_ concentration in computationally simulated P, a peak was observed at t* = 0, and its value was approximately 10^4^ ppm. Subsequently, a monotonic decrease mode occurred until the end of the simulation. This mode transition is similar to the measurement result for the P sensor, although the peak is four times larger than the measurement value, and its decrease is locally (t*~1) nonmonotonic. The simulation and measurement results differed owing to the uncertainties of the initial and boundary conditions in the simulation, mimicking the experimental setup. For example, the airflow through the small gaps between the wall, door, and curtains, the flow direction of the air conditioning devices, and the working staff in the test room cannot be accurately considered in the 3D model. However, the overall trend observed in the measurements is well reproduced in the numerical simulation.

**Figure 6:**
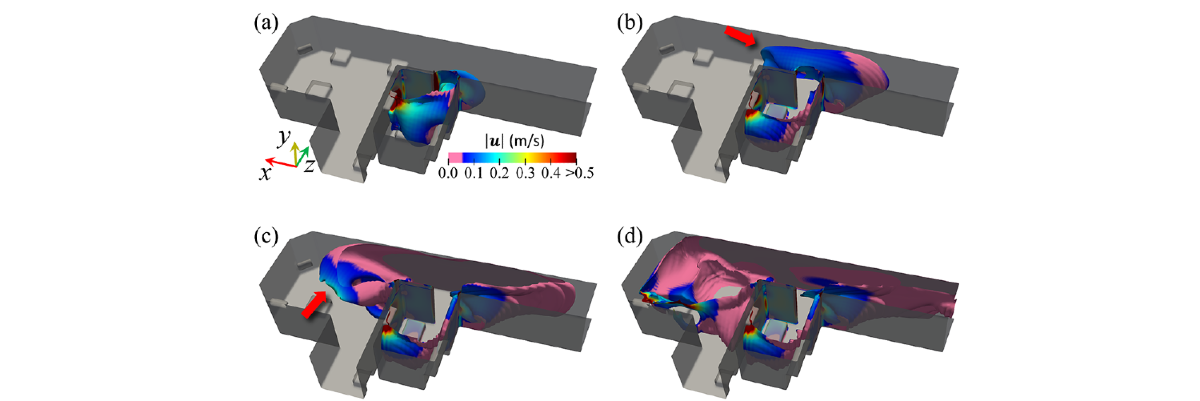
Temporal evolution of the carbon dioxide concentration isosurfaces at 1000 ppm at t* = (a) 0.0, (b) 0.9, (c) 2.8, and (d) 8.4 minutes. The local color of the isosurface represents the local velocity magnitude in m/s (rainbow: high-velocity region, pink: low-velocity region).

Figure 6 provides instantaneous snapshots of the CO_2_ isosurface at 1000 ppm at t* = 0.0, 0.9, 2.8, and 8.4 minutes. A high concentration of CO_2_ was observed throughout the private room when the CO_2_ generation was cut off at t* = 0. The maximum CO_2_ concentration was 8.8 × 105 ppm. Owing to turbulent and molecular diffusion effects, the maximum CO_2_ concentration was monotonically reduced to 2.9 × 10^5^ ppm (t* = 0.9 min; Figure 6), 5.1 × 10^4^ ppm (t* = 2.8 min; Figure 6), and 1.11 × 10^4^ ppm (t* = 8.4 min; Figure 6). Furthermore, the region with a CO_2_ concentration >1000 ppm (high-concentration zone), which is substantially greater than the background value, *C_0_*_,_ is spread across the day room and corridor. In particular, the high-concentration zone occupies the corridor in front of the private room at t* = 2.8 minutes. The local fluid velocity, overlaid on the CO_2_ isosurface, shows that the leading edge of the CO_2_ isosurface moves toward the day room (red arrows in Figure 6) and produces a relatively high velocity. Therefore, large-scale flow dictated the spread of the gas mixture containing infectious aerosols rather than the molecular diffusion process.

## Discussion

### Principal Findings

The CO_2_ tracer gas method was used in 6 areas where airborne infection was suspected, and the ACR was successfully calculated using the primary measurement. On the basis of these results, the ventilation rate in each room at the time of the outbreak met the MHLW guidelines and did not correspond to poorly ventilated space. However, the secondary measurement revealed advection from the private room to the day room by visualizing the CO_2_ tracer gas. The overall trend in gas concentrations detected via secondary measurements agreed well with the results of the numerical simulation. Hence, given that the person who initiated the infection occupied the private room on the day of the infection and that several occupants were gathered in the day room, it was postulated that the infectious aerosol was transmitted by this airflow. These findings confirm the usefulness of the CO_2_ tracer gas method for estimating the ACR and visualizing airflow.

The experimental data confirmed that the opening of windows in this facility promoted natural ventilation and significantly improved the ACR from 2.2 to 6.2 times, thus satisfying the CDC criteria. These findings prompted the facility staff to improve their operations by opening windows when appropriate. Furthermore, in this facility, regular nursing bathrooms were equipped with jalousie windows to ensure the privacy of residents. Therefore, opening windows for additional ventilation does not negatively affect privacy. However, in many older adult care facilities, opening windows is avoided because of concerns regarding dangerous behavior. Residents diagnosed with behavioral and psychological symptoms of dementia can attempt to use the window to leave the facility. Therefore, opening windows as a measure to improve air ventilation should be conducted when considering these safety aspects. For example, a simple locking mechanism can be installed to prevent windows from opening beyond a certain point, thereby effectively ensuring safety and ventilation. In addition, future studies should clarify the effects of open windows on heating and cooling.

The results of the secondary measurements discussed in Figures 5 and 6 suggest that aerosols can be advected between different rooms by the relatively strong fluid flows created by ventilation fans and air conditioning devices. The required ventilation volume (per hour) was determined based on the capacity of the room. For smaller rooms, such as the private or shared rooms in this study, the required ventilation volume is smaller, and vice versa for larger rooms, such as the day room. Therefore, in open or semiopen buildings, where multiple rooms are spatially connected, such as the present facility, advective flows from private to common areas may exist, and adhering to the ventilation volume standard for each room individually may not be sufficient to prevent mass infection outbreaks, as in the present case. This is a challenge faced by many nursing homes where private and common spaces are directly or indirectly connected. However, the facility learned about the risk of aerosol advection from the private room to the day room and subsequently took steps to prohibit its use of this private room. Future facility modifications are planned to reverse the pressure difference by improving the ventilation system to limit or prevent aerosol leakage.

### Limitations

We found that local flows could advect infectious aerosols that could not be predicted solely by the ACR of the individual rooms. This finding was based on a combination of experimental (CO_2_ tracer) and numerical (CFD) examinations, which were performed at this facility. Therefore, a limitation of this study is that it infers the likelihood and route of airborne transmission only from such circumstantial evidence. In the future, it will be possible to trace the order of viral transmission and the route of infection more directly for each individual by analyzing the entire genome of infected people at the time of an outbreak. Furthermore, because various hospitals and offices have video surveillance cameras and entry or exit records, the combination of these security logs, epidemiological data, and 3D models will enable a clear reconstruction of the infection scenario. In addition, a digital contract tracing system [29] and a web-based smartphone app tenant management tool [30] would be useful in evidence building.

Another limitation is the seasonal reproducibility. April 2020, when the outbreak was first reported, was in spring; August 2021, when the experiment was conducted, was in summer. Although mechanical conditions, such as window opening and closing, ventilator, and air conditioner operating conditions, could be reproduced, the amount of buoyant ventilation owing to the difference in indoor and outdoor temperatures, especially when windows were opened, would have varied with the season. Although the effect of buoyant ventilation by season on natural ventilation is another important topic, and a detailed analysis by CFD is desirable, it is outside the scope of this study.

Furthermore, although this study analyzed cases in which the infection spread locally, the results did not indicate a general causal relationship between ventilation volume and infection rate. Future field studies and meta-analyses of a large number of outbreak cases will clarify whether there is a correlation between the amount of ventilation and the risk of COVID-19 infection.

### Comparison With Prior Work

Anderson et al [31] determined that typical older adult care facilities may be vulnerable to COVID-19 because they are designed to promote social interaction and collaboration among residents via common spaces (eg, day rooms and areas for social activities) and hallways without partitions [32,33]. This aspect is important for residents’ social interactions and daily monitoring by staff. Furthermore, in Japan, the deregulation of the Law for Partial Revision of the Building Standards (enacted on September 25, 2018), which exempted the floor area of common corridors from the calculation of the floor area ratio for nursing homes, may have provided an impetus for the active use of corridors as common relaxation areas. However, from the perspective of infection control, there is room for improvement in these open-plan architectural guidelines. With these precedents, practical guidelines should be formulated specifically to address the operational patterns of older adult care facilities. As an example of a temporary guideline during a pandemic, downwind transmission can easily be prevented by discontinuing the use of private rooms close to common rooms in older adult care facilities. The excessive installation of vinyl partitions may also contribute to mass infection [3] due to the stagnation of fluid flow, which is essential for active ventilation. Therefore, care should be taken when designing partitions such that they do not interfere with ventilation. A more quantitative measure would be recommended to monitor the pressure difference between the room and hallway [34], as recommended in health care settings. If the possibility of downwind transmission created by the pressure difference becomes apparent, other measures (eg, transparent partitions or air curtains) can be taken to prevent large-scale airflow, such as that observed in the present physical or fluid-dynamic numerical simulation. However, these measures must consider accessibility and visibility to ensure residents’ quality of life.

### Conclusions

In this study, a real-world mass infection outbreak, which occurred in an older adult care facility, was simulated experimentally and numerically to investigate the controlling factors and quantify the effectiveness of various natural ventilation settings using the ACR, assuming that airborne transmission has occurred. Using the CO_2_ tracer gas method, we determined that the low-cost intervention of opening windows could improve the ventilation frequency by a factor of 2.2 to 5.7. This implies that advective fluid flows are key to controlling the spread of zones with high CO_2_ concentrations. Therefore, this CO_2_ tracer gas method, implemented with dry ice and a sensor, can quantify the ACR and airflow and contribute to the prevention of recurrent airborne infections. Moreover, this method can be performed within a relatively short time, even in the presence of patients or residents.

A numerical simulation was performed to obtain the spatiotemporal evolution in such high CO_2_ concentration zones under conditions similar to those of the present experiment. The development of zones with high CO_2_ concentrations occurred in the first few minutes. Furthermore, the leading edge of such zones toward the day room, where multiple residents gather for activities, yields a relatively high fluid velocity, suggesting that large-scale advective flow dictates the spread of such high CO_2_ concentration zones. These results suggest that secondary infections could occur because of aerosol advection driven by a large-scale flow topology, even if the ventilation is sufficient. Furthermore, this phenomenon may be influenced by architectural design specific to typical older adult care facilities.

To prevent or deter outbreaks of mass infections in older adult care facilities, policies for guidelines on architectural design and reviews of related laws will be necessary, considering both the quality of life of the residents and suppression of large-scale flow toward communal areas. In addition, quantitative studies and interventions are required to avoid downwind contamination of existing buildings.

## Appendix

Multimedia Appendix 1 Time course of the emergence of infected patients in April.

## Abbreviations

ACR: air change rate
CDC: Centers for Disease Control and Prevention
CFD: computational fluid dynamics
GLMM: generalized linear mixed model
CO_2_: carbon dioxide
K: TR-76Ui sensor
MHLW: Ministry of Health, Labor and Welfare
P: SCD-30 sensor
PCR: polymerase chain reaction
WHO: World Health Organization

## Data Availability

The data supporting the findings of this study are available from the corresponding author, YI, upon reasonable request.
